# Repeated (*S*)-ketamine administration ameliorates the spatial working memory impairment in mice with chronic pain: role of the gut microbiota–brain axis

**DOI:** 10.1080/19490976.2024.2310603

**Published:** 2024-02-08

**Authors:** Yubin Jiang, Xingming Wang, Jiawei Chen, Yibao Zhang, Kenji Hashimoto, Jian-Jun Yang, Zhiqiang Zhou

**Affiliations:** a Department of Anesthesiology, Nanjing Jinling Hospital, The First School of Clinical Medicine, Southern Medical University, Guangzhou, China; b Department of Anesthesiology, Pain and Perioperative Medicine, The First Affiliated Hospital of Zhengzhou University, Zhengzhou, China; c Department of Anesthesiology, Affiliated Jinling Hospital, Medicine School of Nanjing University, Nanjing, China; d Department of Anesthesiology, Jinling Clinical Medical College of Nanjing Medical University, Nanjing, China; e Division of Clinical Neuroscience, Chiba University Center for Forensic Mental Health, Chiba, Japan

**Keywords:** Chronic pain, spatial working memory, hippocampus, gut–brain axis, gut microbiota, (*s*)-ketamine

## Abstract

Chronic pain is commonly linked with diminished working memory. This study explores the impact of the anesthetic (*S*)-ketamine on spatial working memory in a chronic constriction injury (CCI) mouse model, focusing on gut microbiome. We found that multiple doses of (*S*)-ketamine, unlike a single dose, counteracted the reduced spontaneous alteration percentage (%SA) in the Y-maze spatial working memory test, without affecting mechanical or thermal pain sensitivity. Additionally, repeated (*S*)-ketamine treatments improved the abnormal composition of the gut microbiome (β-diversity), as indicated by fecal 16S rRNA analysis, and increased levels of butyrate, a key gut – brain axis mediator. Protein analysis showed that these treatments also corrected the upregulated histone deacetylase 2 (HDAC2) and downregulated brain-derived neurotrophic factor (BDNF) in the hippocampi of CCI mice. Remarkably, fecal microbiota transplantation from mice treated repeatedly with (*S*)-ketamine to CCI mice restored %SA and hippocampal BDNF levels in CCI mice. Butyrate supplementation alone also improved %SA, BDNF, and HDAC2 levels in CCI mice. Furthermore, the TrkB receptor antagonist ANA-12 negated the beneficial effects of repeated (*S*)-ketamine on spatial working memory impairment in CCI mice. These results indicate that repeated (*S*)-ketamine administration ameliorates spatial working memory impairment in CCI mice, mediated by a gut microbiota – brain axis, primarily through the enhancement of hippocampal BDNF – TrkB signaling by butyrate.

## 1 Introduction

Chronic pain frequently co-occurs with memory impairment,^1–5^ with about two-thirds of chronic pain patients experiencing deficits in working memory.^6^ These impairments in working memory pose significant management challenges, often leading to decreased quality of life.^7^ Despite this, the precise mechanisms behind working memory impairment in patients with chronic pain remain largely unclear.

Clinical studies have shown that the anesthetic (*R,S*)-ketamine can produce robust antidepressant actions in individuals with treatment-resistant major depressive disorder (MDD).^8–15^ Notably, (*S*)-ketamine, (*S*)-enantiomers of (*R,S*)-ketamine, has been reported to have rapid and sustained antidepressant efficacy in treatment-resistant MDD.^14,16–18^ Beyond its antidepressant properties, (*R,S*)-ketamine might also ameliorate cognitive impairment in patients with MDD.^12,19–21^ However, it is yet to be determined whether (*S*)-ketamine can enhance working memory in either patients or animal models with chronic pain.

Growing preclinical evidence suggests a significant role of the gut microbiome and its metabolites, particularly short-chain fatty acids (SCFAs) such as acetate, propionate, butyrate, in the effects of (*R,S*)-ketamine and its enantiomers in rodent disease models.^12,22–27^ These SCFAs, essential in gut microbiota-brain communication, have been shown to improve cognitive impairment in a rat model of chronic postoperative pain.^22,28,29^ Based on these findings, we hypothesize that the gut microbiota and SCFA-mediated pathways contribute to the beneficial effects of (*S*)-ketamine on spatial working memory in mice with chronic pain.

This study aims to investigate whether (*S*)-ketamine can alleviate spatial working memory impairment in a mouse model of chronic constriction injury (CCI). First, we evaluated the effects of (*S*)-ketamine on spatial working memory using the Y-maze test and analyzed changes in gut microbiome composition through 16S rRNA fecal sample. We then explored the impacts of fecal microbiota transplantation (FMT),^30^ and SCFA supplementation on spatial working memory. Finally, we assessed the role of the brain-derived neurotrophic factor (BDNF)-TrkB signaling pathway in mediating (*S*)-ketamine’s beneficial effects on spatial working memory impairment in CCI mice, considering the pathway’s established connection with (*S*)-ketamine’s other actions.^26,31–33^

## 2 Materials & methods

### 2.1 Animals

Adult male C57BL/6J mice (22–24 g, 7–8 weeks old) were purchased from the Model Animal Research Center of Nanjing University, Nanjing, China, and housed four or five per cage under controlled temperature (22°C) and a 12 h/12 h light/dark cycle (lights on at 7:00 AM) with food and water available *ad libitum*. All mice were allowed one week to acclimatize to the laboratory environment before the experiments. All experimental procedures were conducted in accordance with the Guide for the Care and Use of Laboratory Animals of the National Institutes of Health, USA, U.K Animals (Scientific Procedures) Act, and National Research Council Guide for the Care and Use of Laboratory Animals. All efforts were made to minimize suffering and to reduce the number of mice used in the experiments. All results were reported in accordance with ARRIVE guidelines.

### 2.2 Reagents

(*S*)-Ketamine hydrochloride was purchased from Hengrui Medicine Co. Ltd. (Cat. number: 210511BL, Lianyungang, Jiangsu Province, China), N2-(2-{[(2-oxoazepan-3-yl) amino] carbonyl}phenyl) benzo[b]thiophene-2-carboxamide (ANA-12) from MedChem Express (Cat. number: 219766-25-3, Monmouth Junction, NJ, USA) and sodium butyrate (NaB) from Sigma-Aldrich (Shanghai) Trading Co. Ltd. (Cat. number: V900464, Pudong, Shanghai, China). ANA-12 was dissolved in 5% DMSO + 40% EG300 + 5% Tween-80 + 50% saline before administration, whereas NaB was dissolved in drinking water at 2 mg/ml.

### 2.3 Chronic constriction injury (CCI) of the sciatic nerve

The CCI model was established under aseptic conditions as previously described.^34^ Briefly, the mice were anesthetized with tribromoethanol (0.2 mL/10 g) and the left hind leg was shaved and sterilized with povidone. The sciatic nerve was exposed via blunt dissection through the biceps femoris using a nerve stripper. A 10-mm segment was freed from the adhering tissue at the mid-thigh level and proximal to the trifurcation, and four loose ligatures (5–0 chromic gut, Shandong Boda Medical Products Co., Ltd., Heze, China) were tied around the nerve with 1-mm spacing. The wound was closed by suturing the muscles and skin. Sham surgery was performed by exposing the sciatic nerve as previously described, but without ligation. The mice were then transferred to their home cages and allowed to recover.

### 2.4 Behavioral tests

#### 2.4.1 Open field test (OFT)

Mice were transported to the testing room 1 h in advance to acclimate to their surroundings. The testing room was quiet and dimly lit (30 lx) during the procedure. An individual mouse was placed in the center of a white 40 × 40 cm Plexiglas VersaMax chamber and allowed to explore freely. Movements were recorded, and the total distance traveled in 5 min was calculated automatically using a video tracking system (XR-XZ301, Shanghai Softmaze Information Technology Co. Ltd, Shanghai, China). The test area was cleaned with 70% alcohol between the trials to remove olfactory cues.

#### 2.4.2 Paw withdrawal threshold (PWT)

Mechanical allodynia was tested in a plexiglas chamber with a metal mesh floor using von Frey filaments. The 50% PWT was determined using the up-and-down method.^35^ Briefly, an individual mouse was placed on the mesh floor and allowed to explore the chamber freely for at least 1 h. During the testing phase, a series of von Frey filaments weighing 0.008–2.0 g (Danmic Global, San Jose, CA, USA) was used in ascending order to prick the plantar surface of the hind paw for 3–5 s with sufficient force to induce paw withdrawal, flinching, or paw licking (a positive response) in at least 50% of trials. If a lighter filament induced a < 50% response rate, the next heaviest filament was tested until 50% withdrawal was identified. Each trial was performed at intervals of 5 min, and each weight was repeated three times to obtain the mean value. Finally, the 50% withdrawal threshold was calculated according to the following formula:50% PWT (g) =(10[Xf + kδ])/10 000

#### 2.4.3 Paw withdrawal latency (PWL)

Thermal hyperalgesia was examined using a thermal testing apparatus (IITC Life Sciences, Woodland Hills, CA, USA) in a room maintained at constant ambient temperature. Briefly, an individual mouse was placed on an elevated glass plate and allowed to acclimatize for 1 h before testing. A radiant thermal stimulator was set to 20% of the maximum intensity, and a cutoff of 20 s was focused on the plantar surface of the hind paw through the glass plate. Hind paw lifting and licking were monitored as the signs of thermal pain withdrawal. The time between stimulation onset and cutoff was recorded as the PWL.

#### 2.4.4 Y-maze test of spatial working memory

**Figure 1.**
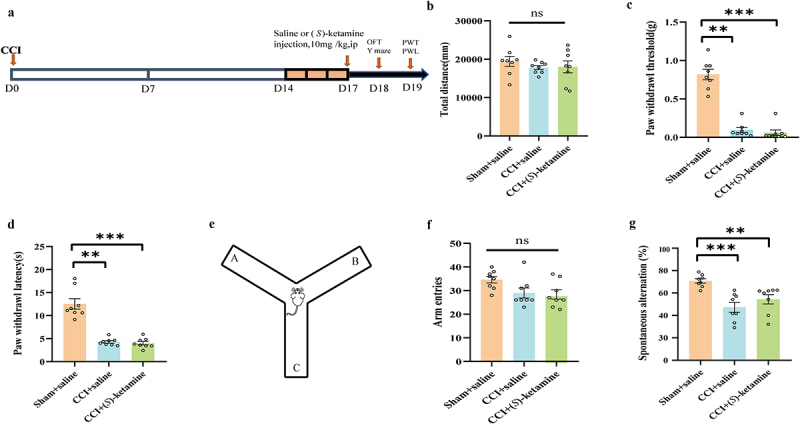
A single-dose injection of (*S*)-ketamine does not ameliorate spatial working memory impairment in CCI mice. a: flowchart and timeline of the experiments. b: the OFT was used to examine locomotor activity (one-way ANOVA: F (2,21) = 0.5423, p = 0.5893). c:PWT (Kruskal-Wallis test, H = 17.428, p = 0.0002). d: PWL (Kruskal-Wallis test, H = 15.58, p = 0.0004). e: schematic of the Y-maze shape. f: number of arm entries in the Y-maze (one-way ANOVA: F (2,21) = 3.410, p = 0.0522. g: spontaneous alternation in the Y-maze (Kruskal-Wallis test, H = 14.77, p = 0.0006). N = 8/group. *p < 0.05, **p < 0.01, ***p <.001. PWT, paw withdrawal threshold; PWL, paw withdrawal latency; OFT, open field test.

The Y-maze used to assess spatial working memory consisted of three arms (each 28 cm long × 6 cm wide × 18 cm high, labeled clockwise as A, B, and C, Figure 1E) diverging at 120° from the central platform. Mice were transported to the test room 1 h in advance to acclimate to their surroundings, and ambient light was maintained at 30 lx throughout. An individual mouse was placed on the central platform and allowed to explore freely for 8 min under video tracking to record the sequence of arm entries. Spontaneous alternation was defined as sequential exploration of all three arms without retracing (ABC, BCA, or CAB, but not BAB, CAC, or CBC). The percentage of spontaneous alternations (%SA) was calculated as follows: (Number of Alternations/[total number of arm entries − 2]) × 100.^36^

### 2.5 Collection of fecal samples from mice

Mice were placed in a clean cage lined with sterilized filter paper, and fresh fecal droppings were collected into sterile dry tubes (minimum 200 mg per tube) using sterilized forceps. Samples from individual mice were mixed with those from the same treatment group, flash-frozen in liquid nitrogen, and stored at − 80°C until analysis.

### 2.6 16S rRNA analysis of feces

The extraction and PCR amplification of fecal genomic DNA were performed by MetWare Biological Science and Technology Co., Ltd. (Wuhan, China). Genomic DNA was extracted using cetyltrimethylammonium bromide (CTAB) or sodium dodecyl sulfate (SDS). Samples were first analyzed by agarose gel electrophoresis to check the purity and concentration of DNA and then diluted to 1 ng/μL in sterile water. Marker rRNAs were amplified using specific primers, the Phusion® High-Fidelity PCR Master Mix with GC Buffer (New England Biolabs), and efficient high-fidelity PCR enzymes.

### 2.7 Measurement of short-chain fatty acids (SCFAs) in feces by gas chromatography-tandem mass spectroscopy (GS-MS/MS)

The SCFA content of fecal samples was determined by MetWare (http://www.metware.cn/) using the Agilent 7890B-7000D GC-MS/MS platform. Stock solutions of standards were prepared at 1 mg/mL in MTBE and stored at −20°C. Immediately prior to analysis, the stock solutions were diluted with MTBE to working concentrations. Fecal samples (20 mg) were weighed and placed in a 2 mL EP tube with 1 mL of aqueous phosphoric acid (0.5% v/v) and a small steel ball. The mixture was ground three times for 10 s each, vortexed for 10 min, ultrasonicated for 5 min, and centrifuged at 12,000 r/min for 10 min at 4°C. A 0.1-mL sample of the supernatant was mixed with 0.5 mL MTBE containing internal standard in a 1.5 mL centrifugal tube and the mixture vortexed for 3 min, ultrasonicated for 5 min, and recentrifuged at 12,000 r/min for 10 min at 4°C. The new supernatant was collected and analyzed by GC-MS/MS using an Agilent 7890 B gas chromatograph coupled to a 7000D mass spectrometer with a DB-FFAP column (30 m length × 0.25 mm i.d. ×0.25 μm film thickness, J&W Scientific, USA).

### 2.8 Antibiotic cocktail treatment and FMT

According to a published protocol,^37^ recipient C57BL/6J mice (male, 8 weeks old) were administered an antibiotic cocktail (ABX, Sangon Bioengineering Mo., Ltd., Shanghai, China) containing ampicillin (1 g/L, Cat. Number:69-52-3), neomycin (1 g/L, Cat. Number:1405-10-3), metronidazole (1 g/L, Cat. Number:443-48-1) and vancomycin (0.5 g/L, Cat. Number:1404-93-9) by oral consumption in drinking water for 14 days, with replacement of the aqueous antibiotic solution every 2 days. This was followed by a washout period of 7 days, during which the animals received sterile tape water to reestablish the gut microbiota prior to FMT.

Fresh mouse fecal pellets from (*S*)-ketamine-treated and saline-treated donor mice were collected, placed into sterilized screw-cap EP tubes, flash-frozen in liquid nitrogen, and stored at − 80°C. To prepare a fecal slurry for FMT, 1 g of feces was homogenized in 10 mL sterile phosphate buffer saline (PBS) and then centrifuged at 2,000 rpm for 5 min at 4°C. The supernatant was transferred to a new sterile tube and stored at − 80°C until use. The recipient mice received 200 µL of freshly prepared fecal slurry or PBS via oral gavage daily for 14 consecutive days.

### 2.9 Western blot analysis

After open field, Y-maze, and pain threshold testing, mice were anesthetized with tribromoethanol (0.2 mL/10 g), and the hippocampus were dissected on ice. The tissues were lysed in RIPA buffer (20 mM Tris-HCl, pH 7.4, 150 mM NaCl, 1 mM EDTA, 1% NP-40, 0.5% sodium deoxycholate, and 0.1% SDS) supplemented with protease and phosphatase inhibitors on ice for 30 min. Lysates were centrifuged at 12,000 × g for 15 min, and the supernatant protein concentration was measured using the BCA method.^38^ Sample protein concentrations were normalized by adding RIPA buffer, and individual samples (40 µg total protein per gel lane) were separated by 10% or 12% sodium dodecyl sulfate polyacrylamide gel electrophoresis (SDS-PAGE), followed by transfer to nitrocellulose membranes. Membranes were blocked with 5% (w/v) skim milk for 1 h at room temperature and then incubated overnight at 4**°**C with the following primary antibodies: anti-BDNF (1:1000, Cat. Number: ab108319, Abcam, Cambridge, UK), anti-histone deacetylase (HDAC) 1 (1:1000; Cat. Number: ab109411, Abcam), anti-HDAC2 (1:1000; Cat. Number: ab32117, Abcam), anti-HDAC3 (1:1000, Cat. Number: ab32369, Abcam), anti-HDAC6 (1:1000, Cat. Number:12834–1-AP, Protein Tech, Wuhan, Hubei, P.R.C) and anti-HDAC8 (1:2000, Cat. Number: ab187139, Abcam). Membranes were rinsed thrice with TBST (5 min per rinse) and incubated with secondary antibodies conjugated to the infrared dye IRdye800 or Irdye700. Blotted membranes were scanned using an Odyssey Infrared Imaging System (LI-COR Biosciences).

### 2.10 Statistical analyses

All data are presented as mean ± SEM and analyzed using SPSS (version 20.0). The normality of distribution was evaluated using the Kolmogorov – Smirnov test and Shapiro-Wilk normality test. All distributions were tested for homogeneity of variance using the Levene’s test. Differences among the groups were assessed using one-way analysis of variance (ANOVA), followed by post hoc Fisher’s least significant difference (LSD) test or two-way AVOVA with post hoc Sidak’s multiple comparisons tests. Non-normal distributions were statistically analyzed using the Kruskal-Wallis test.

The α-diversity of the gut microbiota and relative abundances at different taxonomic classification levels were compared using the Kruskal-Wallis test followed by Dunn’s post hoc test. For β-diversity of the gut microbiota, principal component analysis (PCA) of the operational taxonomic unit (OTU) level, Principal Coordinates Analysis (PCoA), and unweighted UniFrac phylogenetic distance analysis were performed using the analysis of similarities (ANOSIM) function of the R package vegan (2.5.4) Linear discriminant analysis (LDA) effect size (LEfSe) was calculated based on bacterial abundance to identify biomarkers that differed significantly among groups at different taxonomic levels (http://huttenhower.sph.harvard.edu/galaxy/). Only taxa with LDA scores > 3.5 and *p* < 0.05 were considered significantly enriched. The results were visualized using taxonomic bar charts and cladograms. Correlations among butyric acid, Y-maze performance (%SA), hippocampal expression levels of BDNF and HDAC2, and the relative abundance of gut bacteria were analyzed using the Spearman rank test. Statistical significance was set at *p* < 0.05.

## 3 Results

### 3.1 A single-dose injection of (S)-ketamine did not ameliorate spatial working memory impairment in CCI mice

A single dose of (*S*)-ketamine (10 mg/kg) or saline (10 mL/kg) was injected intraperitoneally (i.p.) into sham-operated or CCI model mice (Figure 1A). The total distance traveled in the OFT did not differ significantly between the CCI + saline, CCI + (*S*)-ketamine, and sham + saline groups (Figure 1B). Both PWT (an index used to detect mechanical hyperalgesia) and PWL (an index used to detect thermal hyperalgesia) were significantly higher in the CCI + saline group than in the sham + saline group, confirming the successful establishment of the chronic pain model. However, a single dose of (*S*)-ketamine did not ameliorate mechanical allodynia or thermal hyperalgesia in CCI mice (Figure 1C,D), nor did it enhance spontaneous alterations in the Y-maze test of working memory compared with saline-treated CCI mice (Figure 1G). Furthermore, the number of arm entries did not differ significantly between groups (Figure 1F).

### 3.2 Repeated (S)-ketamine injections ameliorated spatial working memory impairment and hippocampal BDNF downregulation but not mechanical allodynia or thermal hyperalgesia in CCI mice

**Figure 2.**
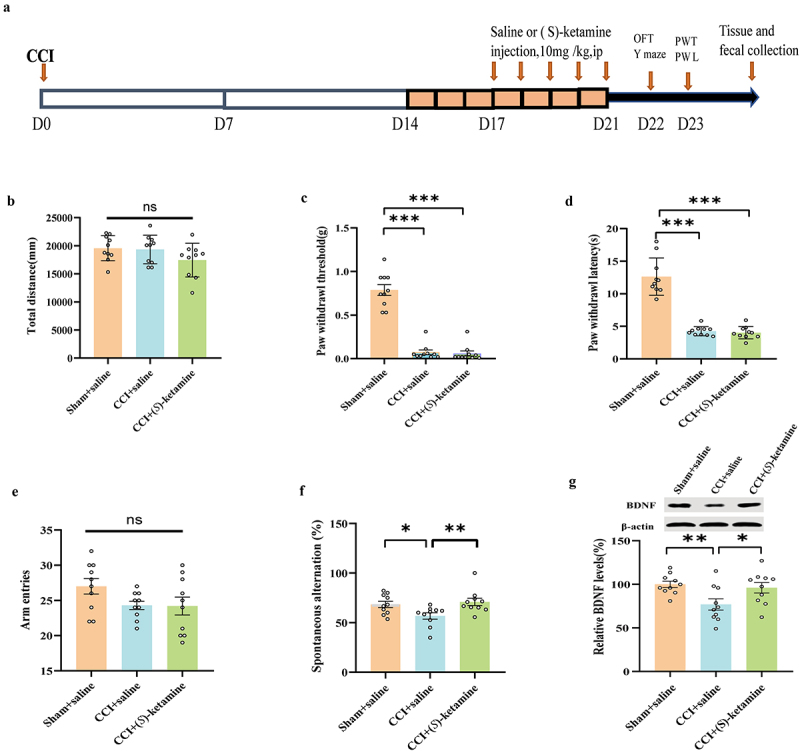
(*S*)-ketamine ameliorated the spatial working memory impairment, BDNF reduction, but not mechanical allodynia and thermal hyperalgesia in CCI mice. a: flowchart and timeline of the experiments. b: the OFT was used to examine locomotor activity (one-way ANOVA: F (2,27) = 1.970, p = 0.1590). c:PWT (Kruskal-Wallis test, H = 20.53; p < 0.0001). d: PWL (Kruskal-Wallis test, H = 19.53, p < 0.0001). e: number of arm entries in the Y-maze (one-way ANOVA: F _(2, 27)_ = 2.368, p = 0.1128). f: spontaneous alternation in the Y-maze (Kruskal-Wallis test, H = 8.309, p = 0.0157). g: BDNF levels in the hippocampus (one-way ANOVA: F _(2, 27)_ = 5.092, p = 0.0133). N = 10/group. *p < 0.05, **p < 0.01, ***p <.001. PWT, paw withdrawal threshold; PWL, paw withdrawal latency; OFT, open field test.

(*S*)-Ketamine (10 mg/kg/day for 5 days) or saline (10 mL/kg/day for 5 days) was injected into sham-operated mice or CCI mice (Figure 2A). The total distance traveled in the OFT did not differ significantly between the CCI + saline, CCI + (*S*)-ketamine, and sham + saline groups (Figure 2B). Both PWT and PWL were significantly lower in the CCI + saline group than in the sham + saline group, confirming the successful establishment of the chronic pain model. Repeated administration of (*S*)-ketamine did not improve mechanical allodynia or thermal hyperalgesia in CCI mice (Figure 2C,D) but did enhance spontaneous alterations in the Y-maze test of working memory compared to saline-treated CCI mice (Figure 2F). Furthermore, the number of arm entries did not differ significantly among the three groups, indicating that the difference in Y-maze performance could not be explained by greater exploratory activity (Figure 2E).

Concomitant with this spatial memory deficit, BDNF expression in the hippocampus was lower in the CCI + saline group than in the sham + saline group, and this reduction was reversed by (*S*)-ketamine administration (Figure 2G). These data suggest that (*S*)-ketamine ameliorates spatial working memory impairment in CCI mice by upregulating BDNF expression in the hippocampus.

### 3.3 Repeated (S)-ketamine injection partially restored normal gut microbiota composition

**Figure 3.**
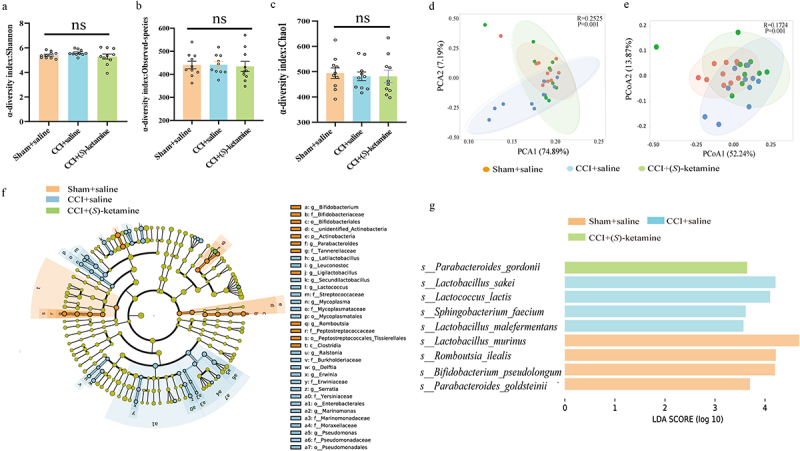
Composition of gut microbiota and LEfSe analysis. a: Alpha-diversity index of Shannon index (Kruskal-Wallis test, H = 2.511, *p* = 0.284975). b: Alpha-diversity index of Observed_OTUs (Kruskal-Wallis test, H = 0.4203, *p* = 0.8105). c: Alpha-diversity index of Chao1 (Kruskal-Wallis test, H = 0.4206, *p* = 0.8103). d: Principal component analysis (PCA) of beta diversity based on OTU levels (ANOSIM, *R* = 0.2525, *p* = 0.001). e: Principal coordinate analysis (PCoA) plot based on unweighted UniFrac distance (ANOSIM, *R* = 0.1724, *p* = 0.001). f: Cladogram (LDA score > 3.5, *p* < 0.05) showing the taxonomic distribution difference among the sham + saline, CCI + saline, and CCI + (*S*)-ketamine groups, indicating a different color region. The inner-outer radiating circles represent taxonomic levels from phylum to genus. g: histograms of the different abundant taxa based on the cutoff value of LDA score (log_10_) > 3.5 and *p* < 0.05, among the three groups; the statistical results are shown in table S1. *N* = 10/group. LDA, linear discriminant analysis; p, phylum; c, class; o, order; f, family; g, genus; s, species.

Fecal samples were collected on day 23 immediately following behavioral testing (Figure 2A) and subjected to 16SrRNA analysis to calculate α-diversity and β-diversity. There were no significant differences in α-diversity, represented by the Shannon index, Chao1 abundance, and Observed_OTUs among the three groups (Figure 3A–), whereas PCA revealed significant separation in the community composition evaluated by ANOSIM (*R* = 0.2525, *p* = 0.001) based on the OTU level (Figure 3D). PCoA was performed based on Unweighted Unifrac distances, and the combination of principal coordinates with the highest contribution was selected for graphical presentation using ANOSIM (*R* = 0.1724, *p* = 0.001) (Figure 3E). Collectively, the repeated administration of (*S*)-ketamine ameliorated the abnormal β-diversity of the gut microbiota in CCI mice.

To identify the gut microbial taxa that differed significantly among the three groups, we further applied the LEfSe algorithm. (*S*)-ketamine significantly altered the phylogenetic distribution of the gut microbiota (Figure 3F,G). Four species-level phylotypes, *Lactobacillus_murinus, Romboutsia_ilealis*, *Bifidobacterium_pseudolongum*, and *Parabacteroides_goldsteinii*, were identified as potential gut microbial markers for the sham + saline group, *Parabacteroides gordonii* was identified as a potential gut microbial marker for the CCI + (*S*)-ketamine group, and four species-level phylotypes, *Lactobacillus sakei*, *Lactococcus_lactis, Sphingobacterium_faecium*, and *Lactobacillus_malefermentans* were identified as potential gut microbial markers for the CCI + saline group (Figure 3F). Collectively, these findings suggest that (*S*)-ketamine ameliorates the abnormal β-diversity of the gut microbiota in CCI mice.

### 3.4 Alterations in gut microbiota composition at different taxonomic levels were associated with fecal SCFA and hippocampal HDAC expression changes

**Figure 4.**
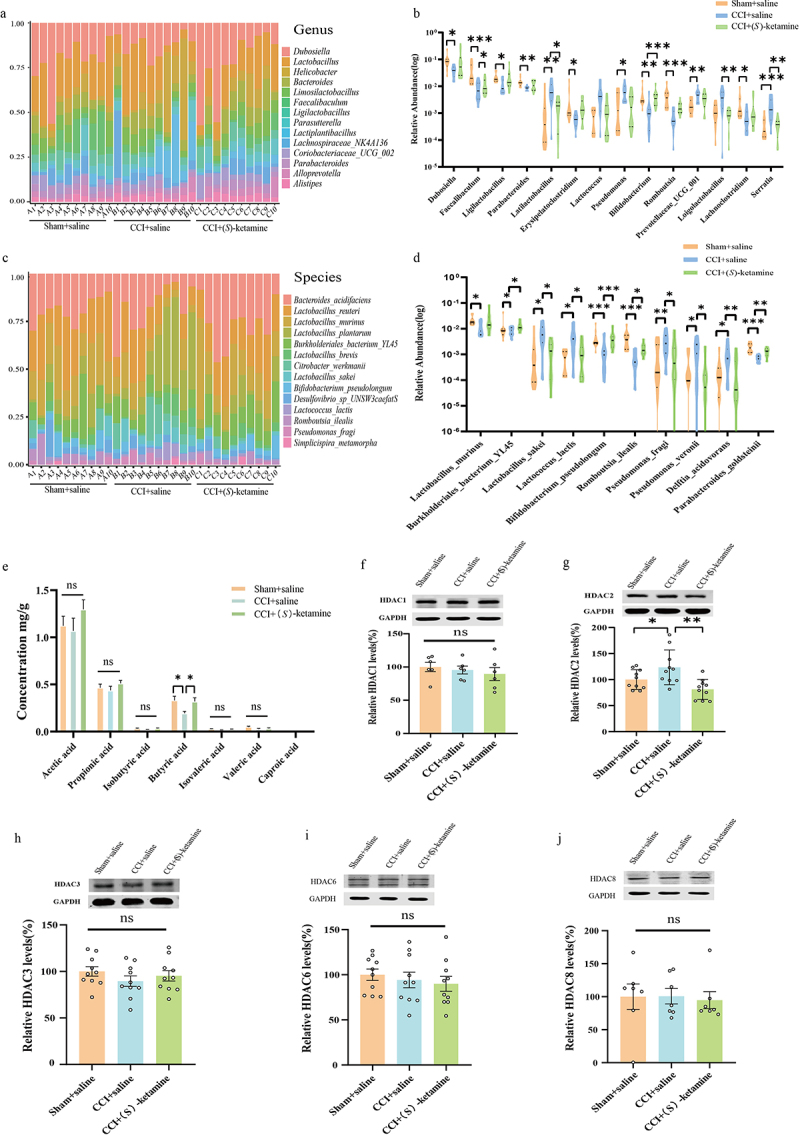
Alterations in the gut microbiota at different levels, including changes in SCFAs in fecal samples and hippocampal HDACs. a, b: genus. The relative abundance distribution and significantly changed bacteria among the sham + saline, CCI + saline, and CCI + (*S*)-ketamine groups are shown in table S2. c, d: species. The relative abundance distribution and significantly changed bacteria among the three groups, the statistical results are shown in table S3. e: SCFA levels in fecal samples from sham + saline, CCI + saline, and CCI + (*S*)-ketamine groups (acetic acid: F_(2, 27)_ = 1.117, *p* = 0.3420; propionic acid: F_(2, 27)_ = 0.9668, *p* = 0.031; isobutyric acid: F_(2, 27)_ = 2.455, *p* = 0.1048; butyric acid: H = 7.235, *p* = 0.027), isovaleric acid: H = 1.86, *p* = 0.394; valeric acid: H = 2.419, *p* = 0.298; caproic acid: H = 6.921, *p* = 0.031). f: the levels of HDAC1 in the hippocampus (one-way ANOVA: F _(2, 15)_ = 0.4738, *p* = 0.6316). *N* = 6/group. g: the levels of HDAC2 in the hippocampus. (one-way ANOVA: F _(2, 27)_ = 7.297, *p* = 0.003), *N* = 10/group. h: the levels of HDAC3 in the hippocampus. (one-way ANOVA: F _(2, 27)_ = 0.9130, *p* = 0.4134), *N* = 10/group. i: the levels of HDAC6 in the hippocampus. (one-way ANOVA: F _(2, 27)_ = 0.4249, *p* = 0.6581), *N* = 10/group. j: levels of HDAC8 in the hippocampus, *N* = 7/group. (Kruskal-Wallis test, H = 0.7644, *p* = 0.7016). *p < 0.05; **p < 0.01; ***p < 0.001. HDAC, histone deacetylase.

The relative abundances of genera *Dubosiella, Faecalibaculum, Ligilactobacillus, Parabacteroides, Latilactobacillus, Erysipelatoclostridium, Lactococcus, Pseudomonas, Bifidobacterium, Romboutsia, Prevotellaceae_UCG_001, Loigolactobacilus, Lachnoclostridium, and Serratia* differed significantly among groups (Figure 4A,B), as did the relative abundances of species *Lactobacillus_murinus*, *Burkholderiales_bacterium_YL45, Lactobacillus_sakei, Lactococcus_lactis, Bifidobacterium_pseudolongum, Romboutsia_ilealis, Pseudomonas_fragi, Pseudomonas_veronii, Delftia_acidovorans*, and *Parabacteroides_goldsteinii* (Figure 4C,D). The fecal butyric acid concentration was significantly lower in the CCI + saline group than in the sham + saline group, and this effect was reversed by (*S*)-ketamine. In contrast, there were no significant differences in the levels of other SCFAs (acetic acid, propionic acid, isobutyric acid, isovaleric acid, valeric acid, and caproic acid) among the three groups (Figure 4E).

Since butyric acid is a histone deacetylase (HDAC) inhibitor,^39^ we measured HDAC1–3, HDAC6, and HDAC8 expression in the hippocampus by western blotting. Hippocampal HDAC2 expression was significantly higher in the CCI + saline group than in the sham + saline group, and this effect was reversed by (*S*)-ketamine administration (Figure 4G). In contrast, there were no differences in the hippocampal expression levels of HDAC1, HDAC 3, HDAC6, or HDAC8 among the groups (Figure 4F–J).

### 3.5 Correlations between the level of butyric acid, BDNF, or the percentage of spontaneous alternation in the Y-maze test, and the significantly differing relative abundance of bacteria at both the genus and species levels

**Figure 5.**
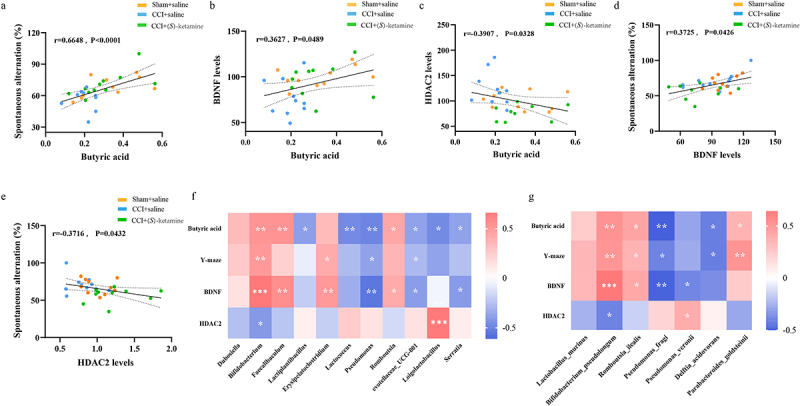
Correlations between the level of butyric acid, BDNF, or the percentage of spontaneous alternation in the Y-maze test, and the significantly differing relative abundance of bacteria at both the genus and species levels.

The %SA in the Y-maze and hippocampal BDNF expression levels were positively correlated with fecal butyric acid concentration (Figure 5A,B), whereas hippocampal HDAC2 expression was negatively correlated with fecal butyric acid concentration (Figure 5C). The hippocampal BDNF expression level was also positively correlated with %SA (Figure 5D), while hippocampal HDAC2 expression was negatively correlated with %SA (Figure 5E). These results suggest that butyric acid synthesized by the gut microbiome may improve spatial working memory in CCI mice by upregulating hippocampal BDNF and downregulating hippocampal HDAC2 expression.

a: Correlation between sodium butyrate and percentage of spontaneous alternation in the Y-maze (*r* = 0.6648, *p* < 0.0001). b: Correlation between sodium butyrate and BDNF expression in the hippocampus (*r* = 0.3627, *p* = 0.0489). c: Correlation between sodium butyrate and HDAC2 expression in the hippocampus (*r* = 0.3907, *p* = 0.0328). d: Correlation between BDNF expression in the hippocampus and percentage of spontaneous alternation in the Y-maze (*r* = 0.3725, *p* = 0.0426). e: Correlation between HDAC2 expression in the hippocampus and percentage of spontaneous alternation in the Y-maze (*r* = 0.3716, *p* = 0.0432). f: Heat map showing the correlation coefficient between bacterial abundance that differs from other groups at the genus level and butyric acid, Y-maze, hippocampal BDNF, and HDAC2 levels (Spearman analysis *r* = 0.35, *p* < 0.05). g: Heat map showing the correlation coefficient between bacterial abundance that differs from other groups at the genus level and butyric acid, Y-maze, hippocampal BDNF, and HDAC2 levels. *p < 0.05; **p < 0.01; ***p < 0.001.

Moreover, fecal butyric acid, %SA, BDNF expression, and HDAC2 expression were correlated with the relative abundance of gut microbial genera that differed significantly among treatment groups. Fecal butyric acid concentration was positively correlated with the relative abundance of *Bifidobacterium*, *Faecalibaculum*, and *Romboutsia*, but negatively correlated with *Lactiplantubacillus*, *Lactococcus*, *Pseudomonas*, *Prevotellaceae_UCG_001*, *Loigolactobacilus*, and *Serratia* abundance, whereas %SA was positively correlated with *Bifidobacterium* and *Erysipelatoclostridium* abundance, but negatively correlated with *Pseudomonas* and *Prevotellaceae_UCG_001* abundance. Hippocampal BDNF expression was positively correlated with *Bifidobacterium*, *Faecalibaculum*, *Erysipelatoclostridium*, and *Romboutsia* abundance but negatively correlated with *Pseudomonas*, *Prevotellaceae_UCG_001*, and *Serratia* abundance. Hippocampal HDAC2 expression positively correlated with *Loigolactobacillus* abundance and negatively correlated with *Bifidobacterium* abundance (Figure 5F). Fecal butyric acid concentration was also positively correlated with *Bifidobacterium_pseudolongum, Romboutsia_ilealis*, and *Parabacteroides_goldsteinii* abundance but negatively correlated with *Pseudomonas_fragi* and *Delftia_acidovorans* abundance. Spontaneous alternation in the Y-maze was positively correlated with *Bifidobacterium_pseudolongum, Romboutsia_ilealis*, and *Parabacteroides_goldsteinii* abundance, and negatively correlated with *Pseudomonas_fragi* and *Delftia_acidovorans* abundance. Hippocampal BDNF expression was positively correlated with *Bifidobacterium_pseudolongum* and *Romboutsia_ilealis* abundance, but negatively correlated with *Pseudomonas_fragi* and *Pseudomonas_veronii* abundance, whereas hippocampal HDAC2 expression was positively correlated with *Pseudomonas_veronii* abundance, but negatively correlated with *Bifidobacterium_pseudolongum* abundance (Figure 5G).

These data suggest that the gut microbiome may regulate Y-maze performance through the gut – microbiota – brain signaling axis involving butyric acid, hippocampal BDNF, and hippocampal HDAC2.

### 3.6 FMT from (S)-ketamine-treated CCI mice can ameliorate the spatial working memory impairment, but not mechanical allodynia and thermal hyperalgesia, in CCI mice

**Figure 6.**
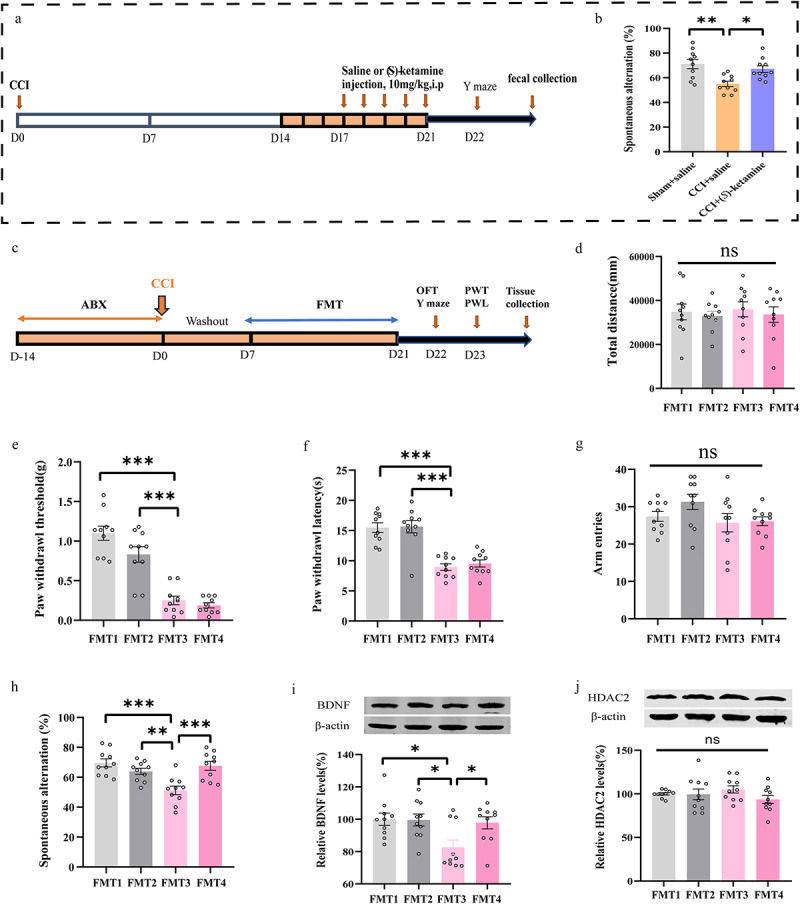
FMT from (*S*)-ketamine -treated CCI mice can ameliorate the working memory impairment, but not mechanical allodynia and thermal hyperalgesia in CCI mice. a: flow chart of fecal sample collection from the donor mice. b: the spontaneous alternation in Y-maze of the donor mice (one-way ANOVA: F _(2, 27)_ = 8.112, *p* = 0.0017). c: the protocol of the FMT experiment. d: OFT: (one-way ANOVA: F _(3, 36)_ = 0.1794, *p* = 0.9097). e: PWT: (one-way ANOVA: F _(3, 36)_ = 36.90, *p* < 0.0001). f: PWL: (one-way ANOVA: F _(3, 36)_ = 22.98, *p* < 0.0001). g: number of arm entries in Y-maze (one-way ANOVA: F _(3, 36)_ = 1.940, *p* = 0.1405). h: the spontaneous alternation in Y-maze (one-way ANOVA: F _(3, 36)_ = 9.594, *p* < 0.0001). i: BDNF levels in hippocampus (one-way ANOVA: F _(3, 36)_ = 4.410, *p* = 0.0097). j: HDAC2 levels in hippocampus (one-way ANOVA: F _(3, 36)_ = 1.124, *p* = 0.3522). *N* = 10/group. *p < 0.05; **p < 0.01; ***p < 0.001. FMT, fecal microbiota transplantation; FMT1, sham + saline-treated sham mice feces; FMT2, sham + saline-treated CCI mice’ feces; FMT3, CCI + saline-treated CCI mice feces; FMT4, CCI + (S)-ketamine-treated CCI mice feces.PWT, paw withdrawal threshold; PWL, paw withdrawal latency; OFT, open field test; CCI, chronic constriction injury.

Fecal samples were collected from donor mice, which included saline-treated sham mice (10 mL/kg/day for 5 days), saline-treated CCI mice (10 mL/kg/day for 5 days) and (*S*)-ketamine- treated CCI mice (10 mg/kg/day for 5 days) (Figure 6A). These samples were confirmed to exhibit the previously observed spatial working memory deficits and reversals, respectively (Figure 6B). The recipient mice received ABX in their drinking water for 14 days, followed by a 7 day period of sterile tap water, before undergoing FMT from the donors of the three aforementioned groups (Figure 6C). The total distance traveled in the OFT was similar across all the four groups of mice (Figure 6D). There were not significant differences in mechanical allodynia and thermal hyperalgesia between the CCI + saline-treated CCI mice feces group and (*S*)-ketamine-treated CCI mice feces groups in mice (Figure 6E,F). While there was no significant difference in arm entries between the CCI + saline-treated CCI mice feces group and CCI + (*S*)-ketamine- treated CCI mice feces group, FMT from (*S*)-ketamine-treated CCI mice significantly improved %SA compared to FMT from saline-treated CCI mice (Figure 6G,H). Western blot analysis also indicated that hippocampal BDNF expression in the CCI + (*S*)-ketamine-treated CCI mice feces group was significantly higher than that in CCI + saline-treated CCI mice feces group (Figure 6I). However, no significant changes were observed in hippocampal HDAC2 levels (Figure 6J). We also performed FMT from (*S*)-ketamine-treated CCI mice feces and only PBS gavage experiment (Supplemental Figure 1). Collectively, these findings suggest that FMT from (*S*)-ketamine-treated CCI mice may alleviate spatial working memory impairment but does not significantly affect mechanical allodynia or thermal hyperalgesia in CCI mice.

### 3.7 Sodium butyrate ameliorated thermal hyperalgesia and spatial working memory impairment, but not mechanical allodynia, in CCI mice

**Figure 7.**
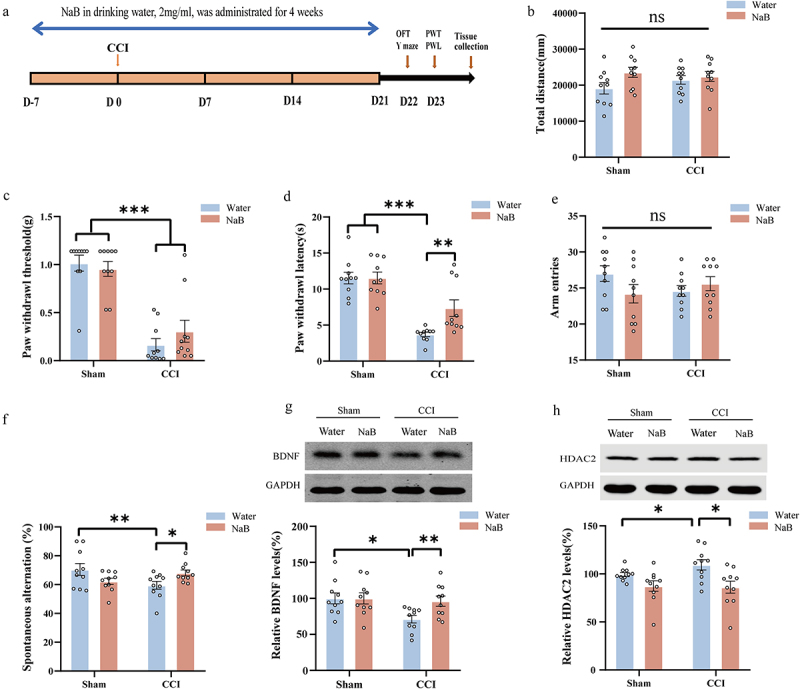
NaB can ameliorate thermal hyperalgesia and working memory impairment, but not mechanical allodynia in CCI mice. a: protocol for the experiment. b: OFT (two-way ANOVA: interaction, F_(1, 36)_ = 1.558, *p* = 0.22; CCI, F_(1,36)_ = 0.1764, *p* = 0.6769; NaB, F_(1,36)_ = 3.672, *p* = 0.0633). c: PWT (two-way ANOVA: interaction, F_(1, 36)_ = 1.334, *p* = 0.2558; CCI, F_(1,36)_ = 74.81, *p* < 0.0001; NaB, F_(1,36)_ = 0.2134, *p* = 0.6469). d: PWL (two-way ANOVA: interaction, F_(1, 36)_ = 4.920, *p* = 0.033; CCI, F_(1,36)_ = 52.77, *p* < 0.0001; NaB, F_(1,36)_ = 4.92, *p* = 0.033). e. Number of arm entries in Y-maze (two-way ANOVA: interaction, F _(1, 36)_ = 3.312, *p* = 0.0771; CCI, F _(1,36)_ = 0.7431, *p* = 0.3944; NaB, F _(1,36)_ = 0.2294, *p* = 0.6349). f: spontaneous alternation in the Y-maze (two-way ANOVA: interaction, F _(1, 36)_ = 8.414, *p* = 0.0063; CCI, F_(1,36)_ = 0.8044, *p* = 0.3757; NaB, F_(1,36)_ = 0.005, *p* = 0.94). g: BDNF levels in hippocampus (two-way ANOVA: interaction, F_(1, 36)_ = 2.121, *p* = 0.1540; CCI, F_(1,36)_ = 1.248, *p* = 0.2714; NaB, F_(1,36)_ = 4.503, *p* = 0.0408). h: HDAC2 levels in hippocampus (two-way ANOVA: interaction, F_(1, 36)_ = 0.9466, *p* = 0.3371; CCI, F_(1,36)_ = 3.881, *p* = 0.0566; NaB, F_(1,36)_ = 5.593, *p* = 0.0235). *N* = 10/group. *p < 0.05; **p < 0.01; ***p < 0.001. PWT, paw withdrawal threshold; PWL, paw withdrawal latency; OFT, open field test; CCI, chronic constriction injury; NaB, sodium butyrate.

To examine the potential signaling function of butyrate in the modulation of working memory impairment by (*S*)-ketamine, three groups of mice were administered exogenous NaB (5 mmol/L) in drinking water for 28 days (Figure 7A). Neither the total distance traveled in the OFT nor the PWT differed between the CCI + water (vehicle) and CCI + NaB groups (Figure 7B,C), but PWL was significantly longer (thermal hyperalgesia reduced) in the CCI + NaB group than in the CCI + water group (Figure 7D). In addition, the%SA was significantly higher in the CCI + NaB group than in the CCI + water group, whereas there was no significant difference in arm entries among the four groups (Figure 7E,F). Furthermore, Western blot analysis showed that hippocampal BDNF expression was higher in the CCI + NaB group than that in the CCI + water group (Figure 7G). In contrast, hippocampal HDAC2 expression was lower in the CCI + NaB group than that in the CCI + water group (Figure 7H). Collectively, NaB appears to ameliorate thermal hyperalgesia and working memory impairment in CCI mice, possibly by downregulating hippocampal HDAC2 and upregulating hippocampal BDNF levels.

### 3.8 TrkB receptor antagonism can block the beneficial effect of repeat (S)-ketamine injection on the spatial working memory impairment in CCI mice

**Figure 8.**
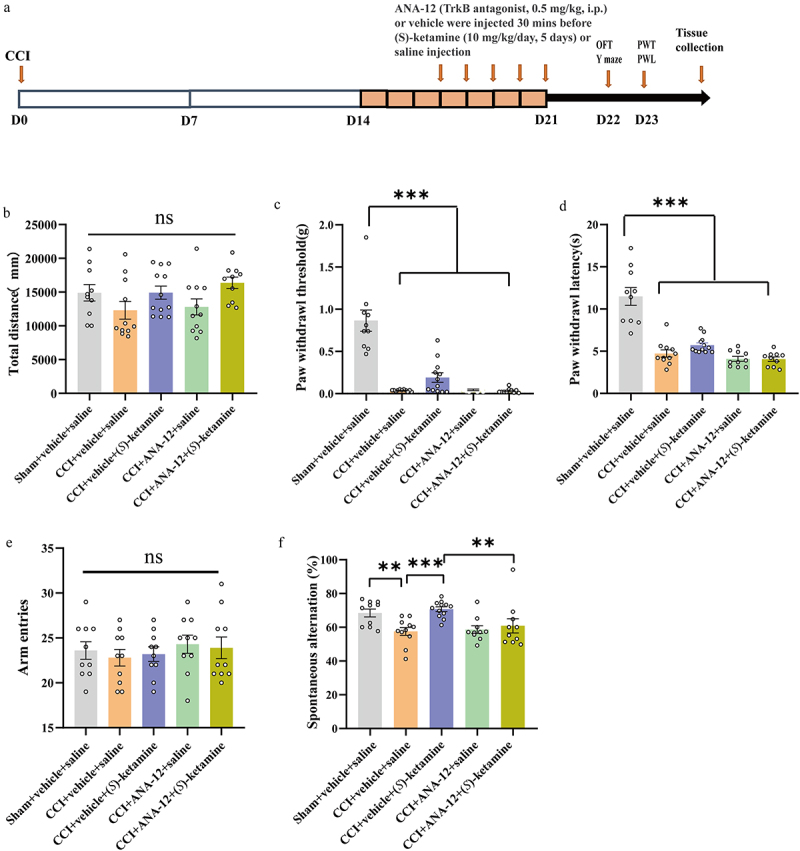
TrkB receptor antagonist can block the beneficial effect of (*S*)-ketamine on the working memory impairment in CCI mice. a: protocol for the experiment. b: OFT (one-way ANOVA: F _(4, 48)_ = 2.204, *p* = 0.0823). c: Kruskal (Wallis test, H = 29.76; *p* < 0.0001). d: PWL (Kruskal-Wallis test, H = 33.49, *p* < 0.0001). e: number of arm entries in the Y-maze (one-way ANOVA: F _(4, 48)_ = 0.3456, *p* = 0.8457). f: spontaneous alternation in the Y-maze (Kruskal-Wallis test, H = 20.55, *p* = 0.0004). *N* = 10/group. *p < 0.05; **p < 0.01; ***p < 0.001. PWT, paw withdrawal threshold; PWL, paw withdrawal latency; OFT, open field test; CCI, chronic constriction injury.

Qiu et al. reported that BDNF can improve postoperative cognitive deficits through TrkB receptor signaling^40^ and we previously found that (*S*)-ketamine increased hippocampal BDNF in CCI mice. Therefore, we examined whether the reversal of spatial working memory impairment in CCI mice was associated with the upregulation of BDNF – TrkB signaling. Mice were injected with the TrkB antagonist ANA-12 (0.5 mg/kg, i.p.) or vehicle 30 min before (*S*)-ketamine (10 mg/kg/day for five days) or saline injection (Figure 8A). There were no significant differences in PWT, PWL, or total distance traveled in the OFT between the CCI + vehicle + (*S*)-ketamine group and the CCI + ANA-12 + (*S*)-ketamine group (Figure 8B–D). However, the%SA was significantly greater in the CCI + vehicle + (*S*)-ketamine group than in the CCI + vehicle + saline group, and this improvement was blocked by ANA-12 administration (Figure 8F). In contrast, the number of arm entries during the Y-maze test did not differ among the five groups (Figure 8E). Collectively, these findings suggest that (*S*)-ketamine may improve spatial working memory impairment in CCI mice through the butyric acid – BDNF – TrkB pathway.

## 4 Discussion

The major findings of this study are as follows: Repeated administration of (*S*)-ketamine significantly improved spatial working memory impairment in chronic pain model (CCI model mice), though it did not affect mechanical allodynia or thermal hyperalgesia. This treatment also partially restored the dysbiosis of the gut microbiota in CCI mice and countered the reduction in microbial butyrate production caused by CCI. Third, it reversed the increase in hippocampal HDAC2 expression and the decrease in hippocampal BDNF expression in CCI mice. Fecal butyrate levels showed a positive correlation with hippocampal BDNF expression and spatial working memory performance in the Y-maze (%SA). These factors, along with the presence of specific gut microbial, were interrelated. Conversely, fecal butyrate, hippocampal BDNF expression, and spatial working memory performance showed negative correlations with hippocampal HDAC2 expression and the presence of certain other gut bacteria deemed harmful. Moreover, FMT from mice treated with repeated (*S*)-ketamine-treated CCI mice and external butyrate administration improved spatial working memory, reduced hippocampal BDNF expression, and increased HDAC2 expression in CCI mice. In addition, the beneficial effects of repeated (*S*)-ketamine treatment in CCI mice were blocked by the TrkB antagonist ANA-12. These findings strongly indicate that (*S*)-ketamine can improve spatial working memory in CCI mice by activating the gut microbiome – brain axis, involving butyric acid and BDNF-TrkB signaling.

The research indicates that (*R,S*)-ketamine has enhance cognitive functions in patients with MDD.^20,21^ Zhang et al.^5^ observed spatial working memory deficits in CCI mice lasting at least 21 days. In contrast, our study found that repeated doses of (*S*)-ketamine (10 mg/kg/day for 5 days), unlike a single dose, effectively reversed spatial working memory impairment in CCI mice. This aligns with previous findings that only repeated (5-day) administrations of (*R,S*)-ketamine (30 mg/kg/day) improve chronic pain-related spatial memory impairment in CCI mice.^41^ Notably, other studies have shown that a single dose of (*S*)-ketamine significantly alleviates cognitive impairment in models of post-stroke chronic stress, lipopolysaccharide exposure, and postoperative conditions.^25,42,43^ The differences between our results and these studies might be due to variations in animal models. In our study, neither single nor repeated (*S*)-ketamine doses reduced pain hypersensitivity in CCI mice. Supporting this, studies have shown that repeated administration of gabapentin (50 mg/kg) or removal of the cuff from the sciatic nerve cuff alleviates pain hypersensitivity but does not improve cognitive impairment in CCI and nerve injury mice.^41,44^ This relationship between cognitive impairment and pain sensitivity thus requires further exploration. Overall, our findings indicate that low-dose (*S*)-ketamine could be a viable treatment for working memory impairment in patients with chronic pain, a hypothesis that should be tested in a randomized placebo-controlled clinical trials.

In this study, neither single nor repeated doses of (*S*)-ketamine administration resulted in improvements in mechanical allodynia or thermal hyperalgesia. Previous research indicates that a single dose of (*R, S*)-ketamine (15 mg/kg) administered intraperitoneally can reduce mechanical allodynia, but its antinociceptive effect mice with chronic pain mice lasts less than 24 hours.^45^ Our pain threshold tests were conducted over 24 hours post-(*S*)-ketamine administration (on day 23 after CCI surgery), which might account for the lack of observed positive effects. In addition to timing, the dosage of (*S*)-ketamine might also significantly influence its antinociceptive properties. While studies on dose-dependent relationship of (*S*)-ketamine’s analgesic effect in animal models are limited, there is accumulating evidence suggesting that the antinociceptive effect of (*R,S*)-ketamine is dose-dependent.^41,46^ Therefore, further research is necessary to fully understand the impact of (*S*)-ketamine on pain sensitivity.

Growing evidence from both clinical and preclinical research suggests that an imbalance in gut microbiota (gut dysbiosis) is linked to cognitive impairment, including working memory deficits. Additionally, the normalization of gut microbial composition is believed to play a role in the therapeutic effects of several drugs.^20,47–49^ For example, alterations in gut microbiota may contribute to the antidepressant-like effect of (*S*)-norketamine, a major metabolite of (*S*)-ketamine, in an inflammatory model of depression.^50^ In our current study, (*S*)-ketamine restored the composition of the gut microbiota in CCI mice. Furthermore, FMT from (*S*)-ketamine-treated CCI mice improved spatial working memory and reduced hippocampal BDNF downregulation. However, this FMT did not reduce the increased expression of hippocampal HDAC2 in CCI mice, indicating that hippocampal HADC2 expression might be influenced by different factors. Overall, these findings suggest that the disruption of the gut microbiota – brain signaling axis plays a crucial role in mediating cognitive deficits associated with certain diseases.

SCFAs are recognized as key mediators in the communication between the gut microbiota and the brain.^28,51^ Our study found that (*S*)-ketamine not only aided cognitive recovery but also reversed the reduction in fecal butyrate levels associated with CCI. Importantly, there was a positive correlation between fecal butyrate concentration and spatial working memory improvement, indicating butyrate’s potential role in influencing the neuro-cellular mechanisms of working memory. Additionally, fecal butyrate levels were positively associated with hippocampal BDNF expression and negatively with hippocampal HDAC2 expression. Butyrate supplementation notably improved thermal hyperalgesia, spatial working memory impairment, and regulated hippocampal BDNF and HDAC2 expression in CCI mice. These data suggest butyrate’s involvement in the neuroplastic processes related to both thermal hyperalgesia and spatial working memory, making it a likely key intermediary in the therapeutic effect of (*S*)-ketamine. Butyrate can cross the blood-brain barrier and influence histone acetylation in the brain,^52^ aligning with our observations of HADC2 downregulation by both (*S*)-ketamine and NaB. The production of butyrate heavily depends on the gut microbiota composition. Interestingly, while butyrate supplementation ameliorated thermal hyperalgesia in CCI mice, neither (*S*)-ketamine nor FMT from (*S*)-ketamine-treated CCI mice produced a similar effect. A previous study reported that the administration of NaB (200 mg/kg and 400 mg/kg, orally) for 14 days significantly reduced hyperalgesia and allodynia in CCI rats.^53^ However, in our study, the NaB intake by mice was considerably low (NaB concentration in water: 2 mg/L). Additionally, the mechanisms of transduction, transmission, and modulation of mechanical and thermal nociceptive sensitivity differ.^54^ These two factors may account for the observed discrepancies. In summary, out data suggest that butyrate enters the brain via circulation and modulates hippocampal HDAC2 and BDNF gene expression or transcription, thereby restoring the neuro-cellular processes essential for working memory.

In our study, the abundances of the genera *Bifidobacterium* and *B. pseudolongum* showed a positive correlation with spatial working memory performance, fecal butyrate concentration, and hippocampal BDNF expression, but a negative correlation with hippocampal HDAC2 expression. Therefore, butyrate produced by these bacteria appears to be a significant mediator of the beneficial effects of (*S*)-ketamine on spatial working memory in CCI mice. Numerous studies have highlighted the role of *Bifidobacterium* species, including *B. pseudolongum*, as butyrate producers with beneficial effects. *Bifidobacterium* supplementation has been shown to improve memory deficits in various mouse models, including middle-aged mice, aged SAMP8 mice, and Alzheimer’s disease model mice.^48,55–60^ Additionally, *Bifidobacterium* supplementation has been reported to enhance stress resilience in a chronic social defeat stress model.^61^ Although the precise functions of *Bifidobacterium* species such as *B. pseudolongum* are not fully understood, they are known to induce anti-inflammatory actions via gut microbiota – brain axis signaling factors, including butyrate.

However, this study had several limitations. Firstly, we did not measure butyrate concentrations in the blood or hippocampus following (*S*)-ketamine administration in CCI mice. Secondly, our assessment of spatial working memory impairment was solely based on the Y-maze test. Finally, the specific microbial species responsible for the beneficial effects of (*S*)-ketamine on spatial working memory impairment in CCI mice were not identified.

## 5 Conclusion

The findings of our study indicate that (*S*)-ketamine improves spatial working memory impairment in CCI mice by engaging a gut microbiota – brain signaling axis. This involves butyrate, which promotes BDNF – TrkB signaling in the hippocampus, and simultaneously suppresses HDAC2-mediated histone deacetylation. Based on these results, further research into effectiveness and safety of (*S*)-ketamine as a treatment for working memory impairments in patients with chronic pain is justified.

## Supplementary Material

Supplemental Material

## Data Availability

The data obtained in the current study are available from the corresponding authors upon reasonable request.

## Data and code availability

The 16s rRNA sequencing data were deposited in the NCBI Sequence Read Archive and are available under the accession number PRJNA951678.
