# Supplementary material for: Repeated (S)-ketamine administration ameliorates the spatial working memory impairment in mice with chronic pain: role of the gut microbiota–brain axis
Source: Gut Microbes. 2024 Feb 8;16(1):2310603. doi: 10.1080/19490976.2024.2310603 (PMC10860353; doi:10.1080/19490976.2024.2310603)
Supplement: Supplemental Material [file KGMI_A_2310603_SM1888.zip › Supplemental_Figure1_caption.docx]

**Supplemental Figure1. FMT from (*S*)-ketamine -treated CCI mice can ameliorate the working memory impairment, but not mechanical allodynia and thermal hyperalgesia in CCI mice**

**A**: Flow chart of fecal sample collection from donor mice. **B**: Spontaneous alternation in the Y-maze of donor mice (unpaired t-test, t = 2.570, P = 0.0199). **C**: Protocol for the FMT experiment. **D**: OFT: (two-way ANOVA: Interaction, F _(1,30)_ = 1.395, P = 0.2468; CCI, F _(1,30)_ = 1.86, P = 0.185; FMT, F _(1,30)_ = 6.968, P = 0.013). **E**: PWT: (two-way ANOVA: Interaction, F _(1, 30)_ = 0.2868, P = 0.5962; CCI, F _(1,30)_ = 86.83, P<0.0001; FMT, F _(1,30)_ = 0.2601, P = 0.6138). **F**: PWL: (two-way ANOVA: Interaction, F _(1, 30)_ = 0.0011, P = 0.9735; CCI, F _(1,30)_ = 132.4, P < 0.0001; FMT, F _(1,30)_ = 4.450, P = 0.0434). **G**: Number of arm entries in Y-maze (two-way ANOVA: Interaction, F _(1, 30)_ = 0.01476, P = 0.9041; CCI, F _(1,30)_ = 0.3037, P = 0.5857; FMT, F _(1,30)_ = 1.551, P = 0.2226). **H**: Spontaneous alternation in the Y-maze (two-way ANOVA: Interaction, F _(1, 30)_ = 2.881, P = 0.1; CCI, F _(1,30)_ = 3.552, P = 0.0692; FMT, F _(1,30)_ = 6.334, P = 0.0174). **I**: BDNF levels in hippocampus (two-way ANOVA: Interaction, F _(1,28)_ = 1.085, P = 0.3065; CCI, F _(1,28)_ = 3.758, P = 0.0627; FMT, F _(1,28)_ = 7.715, P = 0.0097). **J**: HDAC2 levels in hippocampus (two-way ANOVA: Interaction, F _(1,28)_ = 0.8231, P = 0.3720; CCI, F _(1,28)_ = 3.601, P = 0.0681; FMT, F _(1,28)_ = 2.268, P =0.1433). N = 8 / group. *P < 0.05; **P < 0.01; ***P < 0.001. PWT, Paw withdrawal threshold; PWL, Paw withdrawal latency; OFT, open field test; CCI, chronic constriction injury; FMT, fecal microbiota transplantation.
