# Supplementary material for: Repeated (S)-ketamine administration ameliorates the spatial working memory impairment in mice with chronic pain: role of the gut microbiota–brain axis
Source: Gut Microbes. 2024 Feb 8;16(1):2310603. doi: 10.1080/19490976.2024.2310603 (PMC10860353; doi:10.1080/19490976.2024.2310603)
Supplement: Supplemental Material [file KGMI_A_2310603_SM1888.zip › Table S3.docx]

**Supplemental Table 3. Linear discriminant analysis (LDA) score for gut microbiota**

| **Mix taxonomic level** | **Abundance**  **(Log_10_)** | **Group** | **LDA**  **score** | **P**  **value** |
| --- | --- | --- | --- | --- |
| *s__Parabacteroides_goldsteinii* | 4.199385024 | Sham+saline | 3.700220322 | 0.0003 |
| *s__Bifidobacterium_pseudolongum* | 4.573661605 | Sham+saline | 4.198381553 | 0.002 |
| *s__Lactobacillus_murinus* | 5.22711658 | Sham+saline | 4.681177872 | 0.006 |
| *s__Romboutsia_ilealis* | 4.601114853 | Sham+saline | 4.215398348 | 0.002 |
| *s__Lactococcus_lactis* | 4.482923715 | CCI+saline | 4.103710697 | 0.015 |
| *s__Sphingobacterium_faecium* | 3.961126187 | CCI+saline | 3.612830397 | 0.032 |
| *s__Lactobacillus_malefermentans* | 3.903304331 | CCI+saline | 3.569084498 | 0.004 |
| *s__Lactobacillus_sakei* | 4.649654665 | CCI+saline | 4.209891201 | 0.007 |
| *s__Parabacteroides_gordonii* | 4.272751175 | CCI+(*S*)-ketamine | 3.640535364 | 0.017 |

c, class; o, order; f, family; g, genus; s, species.

The cutoff value of LDA score (log10) ​> ​3.5 and P​ < ​0.05 were considered as significant differences. (N =10).
