# Supplementary material for: Repeated (S)-ketamine administration ameliorates the spatial working memory impairment in mice with chronic pain: role of the gut microbiota–brain axis
Source: Gut Microbes. 2024 Feb 8;16(1):2310603. doi: 10.1080/19490976.2024.2310603 (PMC10860353; doi:10.1080/19490976.2024.2310603)
Supplement: Supplemental Material [file KGMI_A_2310603_SM1888.zip › Table S2.docx]

**Supplemental Table S2.** **Statistical analysis of relative abundance of bacteria at species level**

| **Species** | **Sham + Saline (%)** | **CCI + Saline (%)** | **CCI+(*S*)-ketamine (%)** | **Kruskal–Wallis test** |
| --- | --- | --- | --- | --- |
| *Lactococcus_lactis* | 2.148 ± 0.289 | 1.096 ± 0.212^*^ | 2.236 ± 0.723^#^ | H=7.319 , P = 0.026 |
| *Lactobacillus_murinus* | 1.116 ± 0.393 | 0.844 ± 0.092^*^ | 1.259 ± 0.161 | H=6.196 , P = 0.045 |
| *Burkholderiales_bacterium_YL45* | 1.116 ± 0.393 | 0.844 ± 0.092^*^ | 1.259 ± 0.161^#^ | H=6.196 , P = 0.045 |
| *Lactobacillus_sakei* | 0.075 ± 0.019 | 0.544 ± 0.183^*^ | 0.132 ± 0.052^#^ | H =7.346 , P = 0.025 |
| *Bifidobacterium_pseudolongum* | 0.482 ± 0.148 | 0.133 ± 0.061^***^ | 0.423 ± 0.109^##^ | H =11.960, P = 0.003 |
| *Romboutsia_ilealis* | 0.462 ± 0.132 | 0.069 ± 0.017^***^ | 0.171 ± 0.030^#^ | H = 16.635, P<0.001 |
| *Pseudomonas_fragi* | 0.112 ± 0.053 | 0.345 ± 0.100^**^ | 0.152 ± 0.086^#^ | H =7.028 , P =0.030 |
| *Pseudomonas_veronii* | 0.036 ± 0.020 | 0.155 ± 0.053^*^ | 0.026 ± 0.020^#^ | H =6.144, P = 0.046 |
| *Delftia_acidovorans* | 0.032 ± 0.018 | 0.108 ± 0.039^*^ | 0.031 ± 0.018^##^ | H = 7.591, P =0.022 |
| *Parabacteroides_goldsteinii* | 0.193 ± 0.027 | 0.070 ± 0.006^*^ | 0.128 ± 0.014^##^ | H = 17.017, P <0.001 |

The values (relative abundance) are the mean ± S.E.M. (N = 10).

^*^P < 0.05, ^**^P < 0.01, ^***^P < 0.001 compared with Sham + Saline group.

^#^P < 0.05, ^##^P < 0.01, compared with CCI + Saline group.
