# Supplementary material for: Repeated (S)-ketamine administration ameliorates the spatial working memory impairment in mice with chronic pain: role of the gut microbiota–brain axis
Source: Gut Microbes. 2024 Feb 8;16(1):2310603. doi: 10.1080/19490976.2024.2310603 (PMC10860353; doi:10.1080/19490976.2024.2310603)
Supplement: Supplemental Material [file KGMI_A_2310603_SM1888.zip › Table S1.docx]

**Supplemental Table S1.** **Statistical analysis of relative abundance of bacteria at genus level**

| **Genus** | **Sham + saline (%)** | **CCI + saline (%)** | **CCI + (*S*)-ketamine (%)** | **Kruskal–Wallis test** |
| --- | --- | --- | --- | --- |
| *Dubosiella* | 9.032 ± 1.969 | 3.849 ± 0.571^*^ | 9.631± 3.763 | H =6.452, P = 0.04 |
| *Faecalibaculum* | 3.750 ± 1.048 | 0.855 ± 0.200^***^ | 1.123 ± 0.251^#^ | H =11.167, P = 0.004 |
| *Loigolactobacillus* | 2.148 ± 0.289 | 1.097 ± 0.211 | 2.235 ± 0.723^##^ | H =7.184, P = 0.028 |
| *Parabacteroides* | 1.487 ± 0.201 | 0.855 ± 0.054^**^ | 1.136 ± 0.155 | H =7.819, P = 0.02 |
| *Latilactobacillus* | 0.191 ± 0.128 | 0.752 ± 0.200^*^ | 0.146 ± 0.048^##^ | H =10.118, P =0.006 |
| *Erysipelatoclostridium* | 0.349 ± 0.184 | 0.061 ± 0.011^*^ | 0.175 ± 0.048 | H =7.184, P =0.028 |
| *Lactococcus* | 0.076 ± 0.196 | 0.559 ± 0.187^*^ | 0.135 ± 0.055 | H =7.8, P = 0.02 |
| *Pseudomonas* | 0.262 ± 0.099 | 0.694 ± 0.160^*^ | 0.267 ± 0.098 | H =7.278, P =0.026 |
| *Bifidobacterium* | 0.485 ± 0.148 | 0.135 ± 0.062^**^ | 0.424 ± 0.109^###^ | H = 11.862, P=0.003 |
| *Romboutsia* | 0.463 ± 0.132 | 0.068 ± 0.017^***^ | 0.172 ± 0.03 | H =16.68, P <0.001 |
| *Prevotellaceae_UCG_001* | 0.199 ± 0.385 | 0.490 ± 0.089^**^ | 0.360 ± 0.075 | H =7.876, P = 0.019 |
| *Loigolactobacillus* | 0.121 ± 0.030 | 0.407 ± 0.103 | 0.077 ± 0.019^##^ | H=8.825, P=0.012 |
| *Lachnoclostridium* | 0.238 ± 0.092 | 0.072 ± 0.021^*^ | 0.158 ±0.069 | H=5.814, P=0.055 |
| *Serratia* | 0.032 ± 0.013 | 0.191 ± 0.053^***^ | 0.034 ± 0.010^##^ | H=12.284, P=0.002 |

The values (relative abundance) are the mean ± S.E.M. (N = 10). ^*^P < 0.05, ^**^P < 0.01, ^***^P < 0.001 compared with Sham + Saline group.

^#^P < 0.05, ^##^P < 0.01, compared with CCI + Saline group.
