# Supplementary figures and images for: Repeated (S)-ketamine administration ameliorates the spatial working memory impairment in mice with chronic pain: role of the gut microbiota–brain axis
Source: Gut Microbes. 2024 Feb 8;16(1):2310603. doi: 10.1080/19490976.2024.2310603 (PMC10860353; doi:10.1080/19490976.2024.2310603)

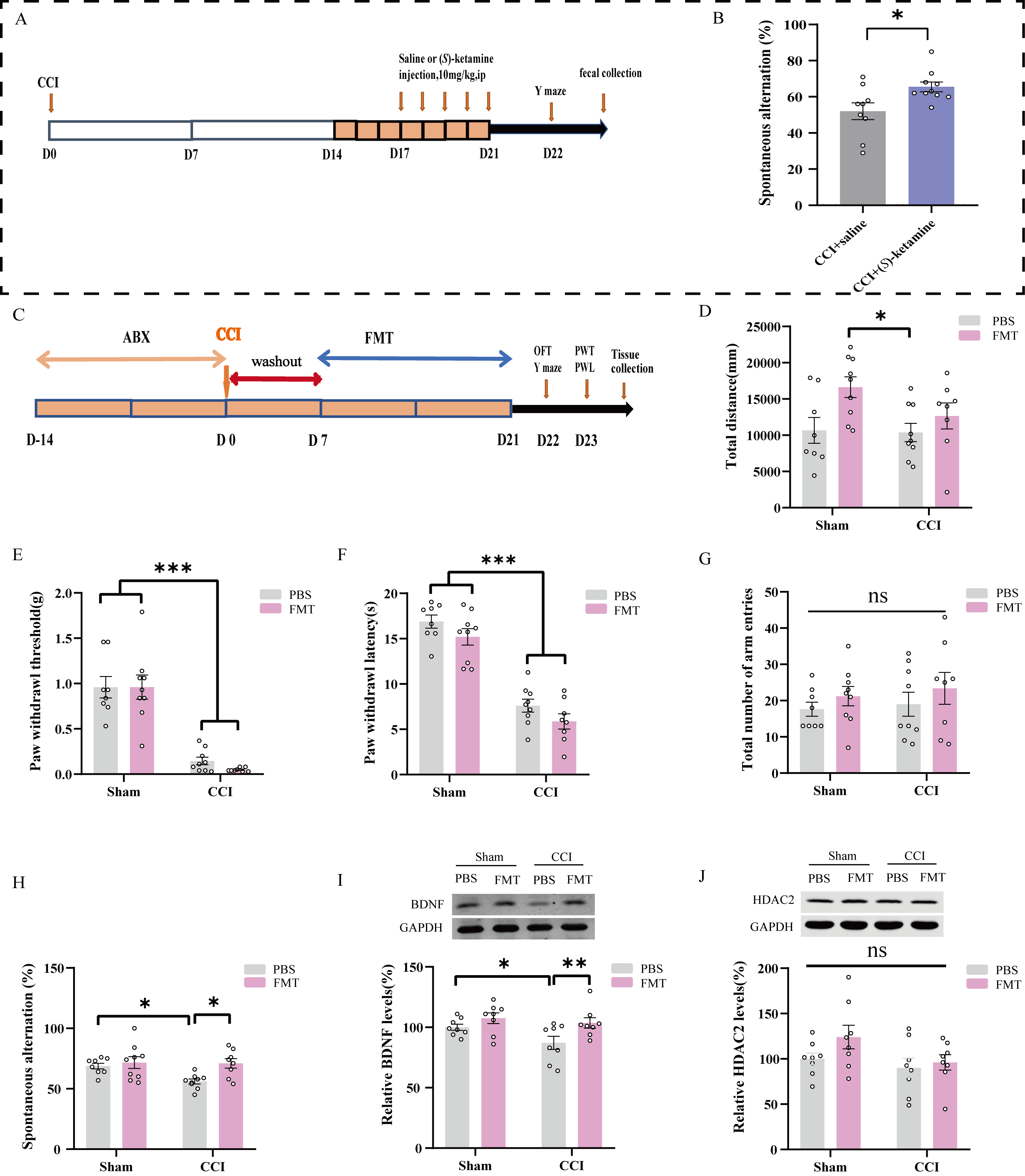

Supplement: Supplemental Material [file KGMI_A_2310603_SM1888.zip › Supplemental_Figure_1.tif]
